# Subtle biases introduced in equity studies through data anonymization

**DOI:** 10.1371/journal.pone.0332441

**Published:** 2025-10-08

**Authors:** Paulo Fazendeiro, Paula Prata, Maria Eugénia Ferrão

**Affiliations:** 1 Instituto de Telecomunicações, Universidade da Beira Interior, Covilha, Portugal; 2 Universidade da Beira Interior, Covilha, Portugal; 3 ISEG Research, ISEG Lisbon School of Economics and Management, Universidade de Lisboa, Lisboa, Portugal; MIT, UNITED STATES OF AMERICA

## Abstract

This work investigates the trade-off between data anonymization and utility, particularly focusing on the implications for equity-related research in education. Using microdata from the 2019 Brazilian National Student Performance Exam (ENADE), the study applies the (ε, δ)-Differential Privacy model to explore the impact of anonymization on the dataset’s utility for socio-educational equity analysis. By clustering both the original and anonymized datasets, the research evaluates how group categories related to students’ sociodemographic variables, such as gender, race, income, and parental education, are affected by the anonymization process. The results reveal that while anonymization techniques can preserve overall data structure, they can also lead to the suppression or misrepresentation of minority groups, introducing biases that may jeopardise the promotion of educational equity. This finding highlights the importance of involving domain experts in the interpretation of anonymized data, particularly in studies aimed at reducing socio-economic inequalities. The study concludes that careful attention is needed to prevent anonymization efforts from distorting key group categories, which could undermine the validity of data-driven policies aimed at promoting equity.

## Introduction

In many nations marked by poverty and inequality, the approach to mitigating these issues involves leveraging empirically grounded knowledge to underpin social policies [1,2]. This specifically entails elucidating how governments secure the fundamental right to quality education for all and implementing measures to diminish inequalities. To fully grasp the transformative role of education [3–5], engaging in scientific research aimed at improving educational equity requires a clear and accurate understanding of what equity means and how it is implemented [6], avoiding any misinterpretations.

For instance, when addressing the reduction of racial or gender inequalities, the way researchers utilize and comprehend group categories becomes crucial, influencing how data analysis fosters the advancement of science for policy. In this context, it may entail researchers accessing microdata or administrative records protected by the General Data Protection Regulation (GDPR). Notably, variables pertinent to research on educational equity are typically safeguarded by the GDPR. In Europe, the GDPR affords special consideration to scientific research to prevent hindrances to research development and align with the goal of establishing a European research area [7]. This study focuses on the educational context, relying on administrative data as evidence. Such analysis constitutes a secondary use of personal data for scientific research purposes. When educational data analysis is classified as a task carried out in the public interest (Article 6(1)(e) GDPR), and appropriate safeguards are implemented in line with Article 89, obtaining explicit consent from students may not be necessary. Under these conditions, the processing of data is considered GDPR-compliant.

Consequently, the regulation and Member States’ laws permit data processing without subjects’ consent, provided that “relevant safeguards are in place.” This necessitates data anonymization by the data controller before sharing with a third party unless specified differently in national laws. The debate over methods ensuring the right to data privacy has persisted for decades [8] featuring methodological contributions by [9–13] and others.

However, using anonymized data for scientific research presents a critical issue, as minority groups tend to be either suppressed or aggregated [14]. This compromises its usefulness for science for policy, especially in endeavors addressing socio-economic inequalities, promoting socio-educational equity, and safeguarding minority rights [15,16].

Data protection and privacy are becoming increasingly significant challenges in the field of Learning Analytics (LA) [17,18]. Modern technology enables the collection of extensive information about educational environments, which can enhance the success of learning outcomes. However, it is crucial to protect the rights and interests of all individuals involved, including students, teachers, and staff. The scoping review presented by Viberg at al. [19] tries to understand how privacy has been defined in LA. It concludes that privacy means different things for the different players in various educational contexts, thus for each case the context and the perspectives of all actors must be taken into account together with applicable regulations. For example, while a student’s personal data may provide educators with valuable insights into the learner’s educational context, the student may perceive this data as sensitive and wish to restrict its disclosure. In short, different stakeholders possess divergent interests, are embedded in asymmetrical power relations, and uphold varying conceptualizations of privacy. The authors highlight that privacy is “one of the most critical success factors for technology use in education … it alone can stop any project or procurement” [19].

The case study about LA and student privacy in K-12 education presented in [20] shows that de-identification is not enough to ensure privacy. It concludes that “Learning analytics and complete student privacy are competing goals that cannot be settled by technology alone” [20].

The main objective of this paper is to investigate whether privacy-preserving data publishing methods compromise the analytical validity of educational datasets for identifying and analysing underrepresented or disadvantaged groups in equity-focused research. To this end, three sub-objectives were set. Firstly, the assessment of the impact of (ε, δ)-Differential Privacy anonymization on the utility of educational microdata used for equity-related research was achieved by the quantitative comparison of clustering structures (using Silhouette, Davies-Bouldin, and Calinski-Harabasz indices) between original and anonymized datasets. Secondly, the evaluation of whether anonymization distorts the representation of minority and underrepresented groups in the data was accomplished by the detection of suppression, misrepresentation, or disappearance of specific group categories (e.g., race, gender, income level) in anonymized clusters. Finally, to determine whether anonymized data preserves the interpretability and reliability of equity-related findings we performed a qualitative and visual comparison (via violin plots) of sociodemographic variable distributions across clusters before and after anonymization.

The results show that even when anonymized datasets retain structural integrity, biases introduced during anonymization can compromise equity-focused analyses. The work highlights the need for domain-informed scrutiny in the interpretation of anonymized data, especially in contexts with policy implications.

The paper is structured as follows. The “Materials and Methods” section describes the dataset, outlines the privacy model used for anonymization, and details the utility model employed to evaluate data usability post-anonymization. The “Results” section presents and interprets the study’s findings, analyzing their broader implications. Then the “Discussion” section debates the limits and potential bias, and finally, the “Conclusion” presents key conclusions to outline potential implications for further research, policy, and practice.

## Materials and methods

Anonymization is an iterative process, wherein an evaluation of the utility of the obtained data should follow each application of a privacy model. Furthermore, the process of anonymization relies on both the nature of the data and the intended purpose of the data analysis. This section starts by describing the datasets under investigation. Next the key strategies for data anonymization are outlined, detailing the chosen privacy model. Finally, the applied utility model is described with details of its operationalization.

### Dataset description

We utilize administrative microdata collected by INEP (Brazilian National Institute for Educational Studies and Research Anísio Teixeira) and publicly available on the INEP’s webpage [21], analyzing the cohort of students who obtained their degree in 2019, with the student as the statistical unit. The datasets used in this study were constructed from the 2019 data of the Brazilian National Student Performance Exam, ENADE 2019, version updated in May 13th, 2021 [21]. The ENADE [22] is conducted annually, offering crucial information to track the quality indicators of higher education. Its results are used by the Ministry of Education (MEC) to monitor and enhance the quality of higher education and to guide regulatory policies. The evaluation is carried out by assessing the academic performance of undergraduate students at the end of their programs, with a focus on the skills, knowledge, and competencies developed during their studies, using standardized examinations scored through Item Response Theory (IRT) models. The variable “grade point average” used in our study was provided by INEP for all students who participated in the examination. In 2019, the number of students involved was 433,930 and the dataset comprises 139 variables. The ENADE dataset is composed of mixed numerical and categorical attributes.

In a previous study [23] the authors utilized this dataset to investigate the utility of data obtained across various anonymization scenarios. The dataset was pre-processed to remove missing, duplicated, and inconsistent data, assuming missing values completely at random, as described in [23]. The resulting dataset, which contains 361,587 records, will be referred to as DS0 or original dataset.

This article considers students’ individual scores, such as their grade point average (gpa1), along with sociodemographic variables, including age, gender, self-declared race/skin color, family income, mother’s and father’s education levels, and region. Two calculated variables were also considered: “years2finish,” representing the number of years needed to complete graduation, and “years_leave,” indicating the elapsed time between secondary and graduated studies.

Table 1 describes the subset of variables considered for this study, showing for each one the label used in the experiments and its range in the original dataset. It is important to emphasize that the variables not included were classified as insensitive, hence having no impact on the anonymization process.

**Table 1:** Selected variables, its labels and range in the original dataset.

Variable	Label	Range
Region	reg	1 = North (N) 2 = Northeast (NE) 3 = Southeast (SE) 4 = South (S) 5 = Central-West (C-W)
Age	age	Between 19 and 86
Gender	gen	M = Male F = Female
Grade point average (specific score)	gpa1	Minimum = 0 Maximum = 93.0
Race/ skin color	race	A = White B = Black C = Yellow D = Pardo E = Indigenous F = Not declared
Father’s education Mother’s education	edu_f edu_m	1 = None 2 = 1st – 5th grade 3 = 6th – 9th grade 4 = Secondary school 5 = Graduation 6 = Post-graduation
Family’s income	inco	1 = Up to 1.5 min. wages 2 = 1.5 to 3 min. wages 3 = 3 to 4.5 min. wages 4 = 4.5 to 6 min. wages 5 = 6–10 min. wages 6 = 10–30 min. wages 7 = Above 30 min. wages
Number of years to finish the graduation	years2finish	Between 3 and 18
Years between high school and college	years_leave	Between 0 and 25

### Privacy model

Statistical disclosure control (SDC) methods have gained significance, particularly following the enactment of the General Data Protection Regulation in the European Union [24]. SDC should be implemented on confidential data before they are published to mitigate the risk of individual data disclosure. This implies removing information and/or modifying the dataset according to a statistical privacy model that enables the quantification of the risk of privacy breaches [25–27]. Concerning privacy models, two main approaches have been pursued: group-based algorithms rooted in the initial k-anonymity method, and randomized algorithms that modify data either through the addition of random noise or by employing random sampling.

The k-anonymity model, introduced by Samarati [28] and Sweney [29] for tabular data, has numerous implementations, most of which are built upon suppression and generalization operations [30]. In the anonymization process, attributes that enable the direct identification of an individual are classified as direct identifiers and subsequently removed. Attributes that, while not directly identifying an individual, allow for association with other datasets, are labeled as quasi-identifiers. The remaining attributes can be further classified as sensitive or non-sensitive. An attribute is considered sensitive if its value should not be disclosed by any adversary with respect to any individual in the dataset; otherwise, the attribute is classified as non-sensitive. Following the application of a k-anonymity algorithm [31], the dataset is considered k-anonymous if the quasi-identifier attributes of any record cannot be distinguished from at least k - 1 other records. Broadly speaking, an adversary has a probability of 1/k to identify a specific record [29].

The most well-known approach for privacy protection through randomized algorithms is differential privacy (DP). Its techniques rely on probabilistic statistical models to measure the extent of disclosure of private information for instances within a dataset. As defined in [32] (p. 9), a randomized function K gives ε-Differential Privacy if for all datasets D1 and D2 differing on at most one element, and all S $\begin{array}{c} \subseteq \end{array}$ Range(K),


$$\begin{array}{c} Pr[\;K(D\text{1})\in \;S\;]\;\leq exp(\varepsilon )\times Pr[\;K(D\text{2})\in \;S\;] \end{array}$$ (1)

The ε parameter, referred to as the privacy budget, serves as a metric for quantifying privacy loss, a smaller ε corresponds to a higher level of privacy protection. For a discussion on determining the value of ε, refer to [33]. A variant of DP is “approximate differential privacy” introduced in [34] and denoted as (ε, δ)-DP. This constitutes a relaxation of pure differential privacy, in which the guarantee provided by Equation (1) holds with probability at least 1−δ. In other words, with probability at most δ, the mechanism may produce an output that does not satisfy ε-DP. As highlighted in [35], it is crucial that the value of δ be very small compared to the number of records when using this variant of DP to ensure meaningful privacy protection.

In DP, privacy protection is not inherent to a dataset but rather a characteristic of the data processing method. Therefore, it can offer stronger privacy protection than syntactic methods such as those based on k-anonymity [36,37]. In this work, the (ε, δ)-DP model is applied to the studied dataset using the SafPub algorithm [38] implemented by the open source ARX - Data Anonymization Tool [39]. The algorithm initiates by randomly sampling the dataset. It subsequently calculates the value of k from the parameters ε and δ and proceeds with k-anonymization. This iterative process is repeated for each potential generalization hierarchy, aiming to find an optimal solution based on a utility model chosen by the user.

Most of DP algorithms are designed to address queries against data in scenarios where the data custodian retains control and does not publish it [40]. Algorithms such as SafPub allow for the use of DP in situations where the objective is to publish the data in an anonymized manner.

### Utility model

The tradeoff between data privacy and data utility when applying privacy-preserving mechanisms has attracted much attention, *cf.* [41], due to the fact that there is a loss of information when any anonymization mechanism is applied to a protected data set. Data utility models and metrics aim at quantifying the quality of the anonymized data for further analysis and modelling purposes. For general analysis usually information loss metrics are more suitable, measuring the similarity between the original and the anonymized data, whereas for specific purposes the data utility is assessed by comparing the accuracy of a certain task using the original and the anonymized data, *cf.* [42].

In the context of preserving privacy and utility, exploratory data analysis using clustering pursues two distinct, yet complementary, goals: (i) to provide a meaningful insight on the overall structure of the data and (ii) to produce a manageable representation of a collection of objects into homogeneous groups [43]. Based on our previous work [15], we propose using cluster validity analysis as a complementary approach to evaluate the effectiveness of the deduced anonymization models.

To search for meaningful insights and groupings that are not achievable with algorithms limited to a single data type we used the K-Prototypes algorithm [44]. It is an extension of the K-Means clustering algorithm that is specifically designed to handle mixed data types. While K-Means is suitable for numerical data, K-Prototypes can work with both numerical and categorical data, making it more versatile for real-world datasets that, as occurs with ENADE data, contain a mix of these types. The distance between a data point and a prototype is calculated using a combination of the numerical distance (for instance the Euclidean distance) for numerical attributes and a dissimilarity measure (for instance the Hamming distance) for categorical attributes. The total distance is a weighted sum of these two distances. Each data point is assigned to the prototype (cluster) closer to it according to the total distance. This step is like the assignment step in K-Means. The prototypes (centroids) are updated based on the mean of the numerical values and the mode of the categorical values in each cluster. This ensures that the prototypes reflect the central tendency of the data points assigned to them.

Compared to K-Modes (restricted to categorical data) and K-Medoids-based methods such as PAM or CLARA (computationally more demanding due to their reliance on pairwise dissimilarities and medoid updates) K-Prototypes offers greater efficiency and scalability which are essential given the size of the ENADE dataset. Alternative approaches such as hierarchical clustering based on Gower distance, density-based methods such as DBSCAN, or probabilistic models like Latent Class Analysis (LCA), were not pursued due to their limited scalability and increased model complexity. On the other hand, K-Prototypes was able to produce interpretable centroids that combine means and modes, enabling straightforward analysis of cluster profiles while maintaining computational tractability for iterative runs over high-dimensional, mixed-type data.

In this context, the anonymization can be seen as a smoothing procedure that increases the probability of discarding small clusters with consequent prejudice on the correct identification of the underlying data structure. It is expected that, by performing the cluster validity analysis on the anonymized dataset and the sequent comparison with the results obtained for the original dataset, the utility is only preserved whenever the underlying structure of the data does not change, hence allowing the correct identification of groups of interest.

Cluster validity is the evaluation of clustering results to find the partitioning that best fits the underlying data preventing the adoption of a partitioning scheme that results in wrong decisions. This assessment can be done in a relative manner by comparing the values of some indices of the clustering structures when the same algorithm with different parameters is applied (a common choice when using partitional clustering is varying the number of clusters, k, to be formed). Generally speaking, an optimal clustering scheme should exhibit good levels of compactness (the distance between members of each cluster should be as small as possible) and separation (the clusters themselves should be far apart from each other). In this work we used as cluster validity indices three indices commonly used in practical applications: Silhouette, Davis-Bouldin and Calinski-Harabasz, cf. [45]. The purpose was to verify if there were major differences in data structure prior and after anonymization. However, as it will be made clear in the next section, this requirement is not enough since, as we know, the output of a clustering algorithm is only a hypothesis on the summarization or explanation of data.

Hence, we performed an extra step in our analysis by identifying (or labeling, if you wish) the corresponding clusters. We computed the inter-cluster distance between each cluster of the original data towards each cluster formed on the fresh anonymized data. The assumption was that the pairs of clusters with smaller inter-cluster distance were indeed one and only one matching cluster (prior and after anonymization). Afterwards we fine-grained our analysis focusing on a set of social-equity attributes to perceive if, despite this structural correspondence, there were (or were not) observable equity-related biases on the anonymized dataset.

As a final remark, please note that we made the studied datasets publicly available in the Open Science Framework repository (https://doi.org/10.17605/OSF.IO/P9M7N). The source code of the anonymization tasks is publicly available as open-source software. The clustering-validity pipeline source code, based on the scikit-learn Python library, was also made publicly available (https://github.com/Farmerinpt/clustering-anonymization-utility). To evaluate the quality of each partition in this work, we selected three indices that are commonly referenced in the literature, i.e., Silhouette, Davis-Bouldin and Calinski-Harabasz. Broadly speaking, these indices estimate the clusters’ cohesion and the clusters’ separation, combining them to produce a quality measure. For Silhouette and Calinski-Harabasz the best partitions correspond to higher values whereas for Davis-Bouldin lower values are better.

## Results

We conducted a data analysis using clustering to examine the impact of data anonymization on the perceived *a posterior* utility of a large dataset. We centered our study on the ENADE dataset specifically on what may happen to the quality of the new data in case it is going to be used for equity studies. For that reason, the following results discuss only equity attributes of the dataset such as gender, race, region, income, and parents’ education.

During the anonymization process, five (ε, δ)-differential privacy scenarios were evaluated. For privacy budget (ε) values of 1, 2, and 3, a fixed δ value of 0.01 was applied. Additionally, for ε equal to 1, δ values of 0.01, 0.001, and 0.0001 were considered to assess the impact of varying privacy guarantees. Among the scenarios analysed, the configuration with ε = 1 and δ = 0.01 yielded the lowest utility loss, with a re-identification risk of 5.2% [23]. Therefore, in this work, we used the dataset obtained through (1, 0.01)-DP anonymization of DS0, as described in [23], and we will refer to it as DS1 or anonymized dataset that has 215,097 records. Empirical distributions for region, gender, race/skin color, parental education, and income are included as supplementary material file for both the original and anonymized datasets (see S1 Table). As anticipated, the distributions of quasi-identifying variables underwent substantial shifts, while those of non-sensitive variables remained largely stable. Overall, approximately 33% of records associated with quasi-identifying variables were removed during the anonymization process.

**Fig 1:**
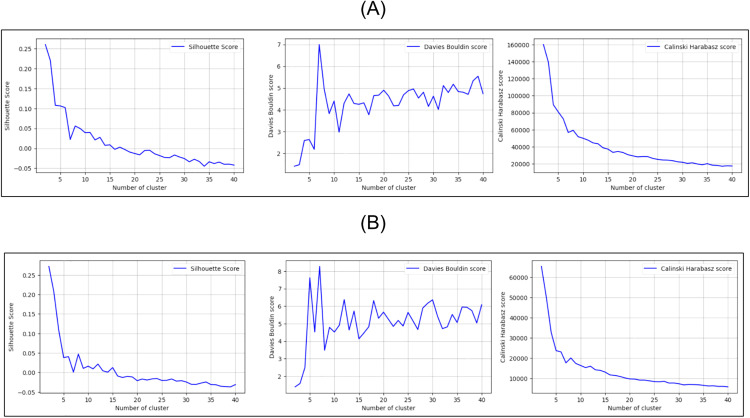
Cluster validity indices Silhouette score, Davies-Bouldin score and Calinski-Harabasz score. (A) Results after clustering the original dataset DS0. (B) Results after clustering the anonymized dataset DS1.

We performed 10 independent runs of the K-prototypes algorithm, each one with the number of groups to be formed (K) ranging from 2 to 40 clusters. The average values for the cluster validity indices of the different runs are presented in Fig 1.

We can say that for the ENADE dataset those measures are consistent both in the original dataset, DS0, and in the anonymized dataset, DS1. The value of K=6 seems to be a fairly good candidate for the number of major chumps of data. Computing a simple inter-cluster distance between the clusters as the distance between their centroids, prior and after anonymization, as depicted in Table 2, it is possible to establish a one-to-one correspondence between the clusters formed in the original dataset and the ones produced after anonymization.

**Table 2:** Centroid inter-cluster distance.

	*o1*	*o2*	*o3*	*o4*	*o5*	*o6*
***a1***	1,766	1,362	1,683	0,932	0,677	2,096
***a2***	1,164	1,695	1,240	0,573	0,572	1,667
***a3***	1,737	2,351	2,044	2,137	1,844	0,181
***a4***	1,632	1,508	1,525	0,935	0,173	1,830
***a5***	1,782	0,679	0,245	1,455	1,629	2,148
***a6***	2,007	1,965	1,516	0,210	0,990	2,172

Labels o1 to o6 refer to the observed clusters prior to the anonymization step.

Labels a1 to a6 refer to the observed clusters after anonymization.

As a matter of fact, we can say that in both situations, apart the need for some reorder, the identified 6 clusters share the description depicted in Table 3.

**Table 3:** Linguistic description of the six clusters.

C1: Low income, high level of education, *gpa1* above average.
C2: High age, average income, low level of education, gpa1 below average.
C3: Income and education slightly above average, gpa1 slightly below average.
C4: High income, high level of education, high gpa1.
C5: Low income, low level of education, gpa1 below average.
C6: Race D (brown), low income, gpa1 below average.

To support the interpretation of the clustering results, the S2 and S3 Tables heatmap tables display the prevalence and lift values of each modality across the identified clusters for the original dataset (see S2 Table) and for the anonymized dataset (see S3 Table). Prevalence refers to the proportion of individuals within a given cluster who exhibit a specific modality (e.g., a certain income level or education category), thereby capturing how common that modality is within the group. Lift, on the other hand, measures the overrepresentation or underrepresentation of a modality in a cluster compared to its frequency in the entire dataset (the cohort). A lift value greater than 1 indicates that the modality is more prevalent in the cluster than expected by chance, while a value less than 1 indicates underrepresentation. The combined heatmap facilitates the identification of characteristic features of each cluster and helps to highlight potential biases introduced during the anonymization process.

A visual inspection of the S2 and S3 Tables allows us to perceive that despite the noticeable correspondence between the clusters prior and after anonymization, in some cases there are changes in the prevalence and lift patterns.

**Fig 2:**
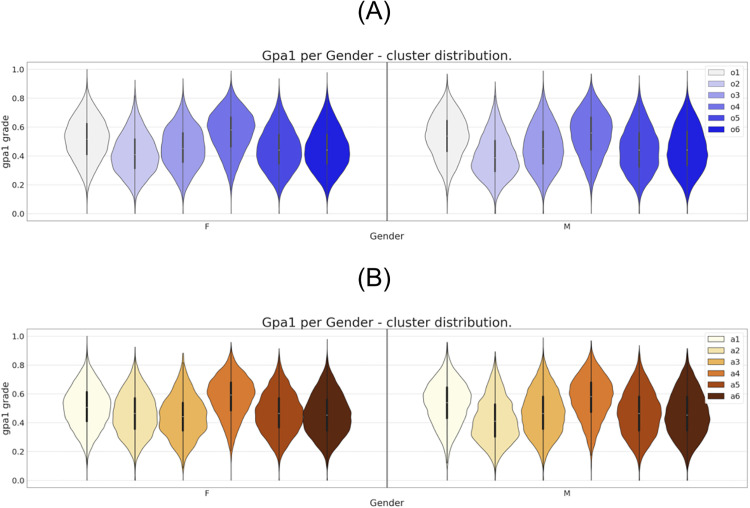
Six clusters distribution of gpa1 for gender attribute. (A) Clustering of the original dataset. (B) Clustering of the anonymized dataset.

**Fig 3:**
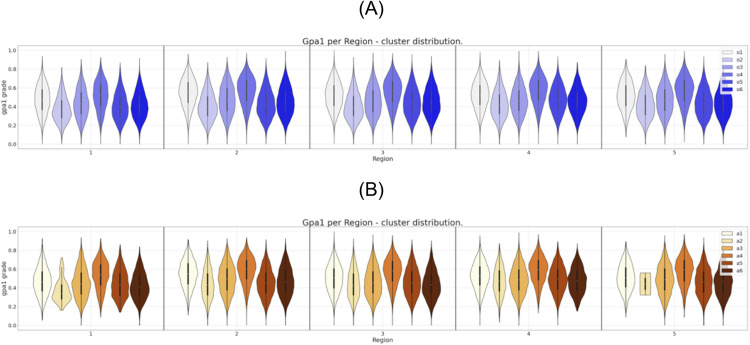
Six clusters distribution of gpa1 for region attribute. (A) Clustering of the original dataset. (B) Clustering of the anonymized dataset.

**Fig 4:**
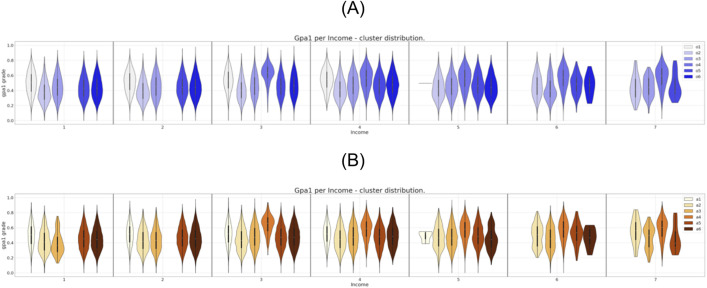
Six clusters distribution of gpa1 for income attribute. (A) Clustering of the original dataset. (B) Clustering of the anonymized dataset.

**Fig 5:**
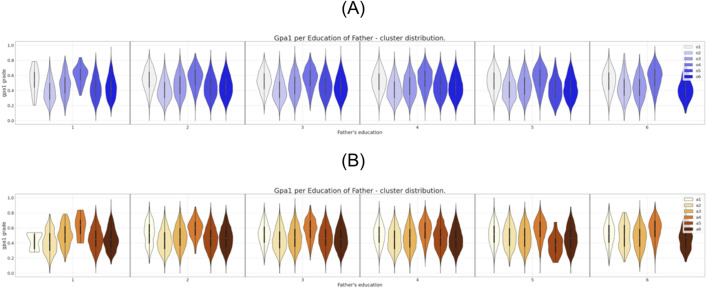
Six clusters distribution of gpa1 for father’s education attribute. (A) Clustering of the original dataset. (B) Clustering of the anonymized dataset.

**Fig 6:**
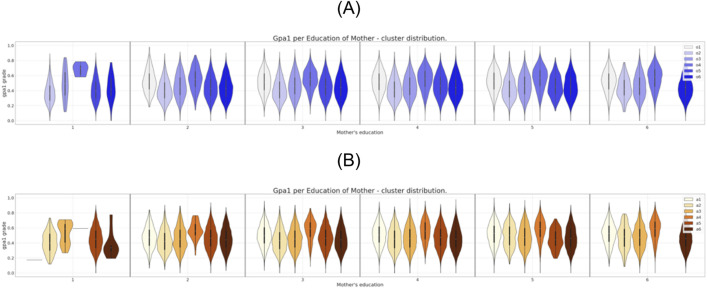
Six clusters distribution of gpa1 for mother’s education attribute. (A) Clustering of the original dataset. (B) Clustering of the anonymized dataset.

**Fig 7:**
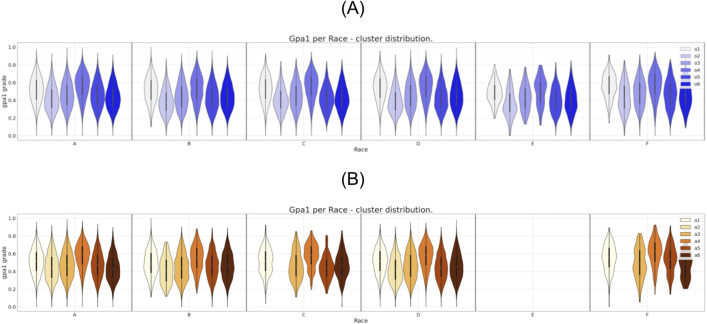
Six clusters distribution of gpa1 for race attribute. (A) Clustering of the original dataset. (B) Clustering of the anonymized dataset.

This correspondence is detailed in the violin plots presented in Figs 2–7. This method of visualizing the distribution of a dataset combines aspects of a box plot and a kernel density plot, providing a comprehensive view of the data’s distribution. It presents a smoothed version of the histogram that represents the gpa1 data distribution, mirrored on both sides of a central line, thus resembling the shape of a violin. It also incorporates in the central line a box plot, indicating the median, quartiles, and potential outliers of the gpa1 values. The width of the “violin” at different values represents the density of the data at those values. Wider sections indicate higher data density, while narrower sections indicate lower data density. Fig 2 presents the violin plot of the six clusters for the gpa1 distribution across gender in each of the six clusters, prior to anonymization, *oi*, and after anonymization, *ai*.

It is possible to observe in Fig 2 the direct match between the gender distributions for the six clusters prior and after the data anonymization. The same correspondence can be observed, eventually with some localized differences, for the set of attributes region, income, and father’s education as can be seen in Figs 3,4, and 5, respectively.

In what concerns the attributes “mother’s education” and “race” (Figs 6 and 7 respectively) the differences in the clusters’ constitution are more notorious than the ones observed for the previous attributes. For instance, comparing Fig 6(a) and (b), we can see that after anonymization one of the clusters cease to have representatives with a low level of mother’s education. Even more serious than that, as can be seen from the comparison between Fig 7(a) and (b), after anonymization every cluster has lost any individual from a given underrepresented race E. In the same Fig. we can see that the cluster “a2” also ceases to have representatives from the races C and F.

By performing cluster validity analysis on the anonymized dataset and comparing it with the results from the original dataset, we find that data utility is preserved only if the underlying structure of the data remains unchanged. This preservation allows for the correct identification of groups of interest. However, this condition alone is not sufficient. The reported results indicate that, for this case study, there are challenges in interpreting the anonymized data, particularly regarding equity-related variables.

## Discussion

While the results presented in this study provide important insights into the impact of anonymization on the utility of microdata for equity-related research, several limitations must be acknowledged. First, the study relies on a single privacy model, (ε, δ)-Differential Privacy. This choice was grounded in prior utility assessments for the same dataset, however, alternative privacy-preserving methods could yield different outcomes in terms of structural distortion or group suppression. Second, the analysis is confined to one national dataset (ENADE 2019), with its specific sociodemographic structure, policy context, and educational system. The generalizability of the findings to other educational datasets or international settings may be limited. Third, the anonymization process was shown to suppress or remove certain underrepresented groups (e.g., individuals from specific racial categories), which may introduce systematic biases, particularly in studies aiming to inform equity policies. Such distortions were qualitatively described, but a formal quantification of their effect on policy modeling was beyond the scope of this study. Moreover, the evaluation of utility was performed in the context of unsupervised learning, focusing on the preservation of cluster structure. Other analytical contexts, e.g., predictive modeling, require different utility metrics, and the observed effects of anonymization could raise a different set of biases.

Lastly, not all variables in the dataset were considered in the analysis; only those deemed sensitive and relevant to equity studies were included. This choice may limit the detection of other distortions in less prominent subpopulations.

## Conclusion

Education’s transformative power calls for scientific research that enhances equity without distorting its meaning or application. In efforts to reduce inequalities, the interpretation and use of group categories by researchers play a critical role in advancing data-driven policy. It goes without saying that statistical disclosure control methods are crucial for protecting confidential data before publication, which involves modifying or removing data – operation supported by a statistical privacy model that quantifies the risk of privacy breaches. However, it is of paramount importance, when the research is conducted over quasi-identifying or sensitive data, to guarantee that the anonymization effort does not tamper the research findings. When anonymization techniques are applied (relying on the incorporation of group-based algorithms in any point of their pipeline) a special care is needed to prevent this risk.

As was demonstrated in this work, even when the overall structure of a clustered anonymized dataset matches the one from the clustered original dataset – apparently revealing the same groups of interest in both cases – there remain differences at the heart of the formed groups that can give raise to difficulties of interpretation and analysis. For instance, what happens when the whole representatives of a minority, underrepresented group, disappear from the new anonymized data or cease to be present in a subset of the anonymized clusters? What measures should be taken? More important than that, what should be done to assure that the anonymization process does not introduce such kind of biases?

In a nutshell, while anonymized datasets may appear to mirror the structure of original datasets, significant differences can emerge within the core of the groups produced by automated unsupervised learning techniques, leading to challenges in interpretation and analysis. The disappearance or underrepresentation of minority groups in anonymized clusters may introduce potential biases resulting from the anonymization process. To address this, it is crucial to develop measures that ensure these biases are minimized, preserving the integrity and inclusivity of the data for accurate analysis and decision-making. Furthermore, regulations concerning the accreditation of scientists and the conditions for accessing microdata required for research in the public interest should strike a careful balance between data protection and the need to advance scientific knowledge in domains critical to humanity.

## Supporting information

S1 Table Empirical distributions. (PDF)

S2 Table Prevalence and lift values for the original six clusters. (PDF)

S3 Table Prevalence and lift values for the six clusters after anonymization. (PDF)
