# Supplementary material for: Subtle biases introduced in equity studies through data anonymization
Source: PLoS One. 2025 Oct 8;20(10):e0332441. doi: 10.1371/journal.pone.0332441 (PMC12507250; doi:10.1371/journal.pone.0332441)
Supplement: S1 Table — (PDF) [file pone.0332441.s001.pdf]

Table S1. Empirical distributions of DS0 and DS1

Variable/Attribute type	Range	DS0 (%)	DS1 (%)
Region / Quasi-identifier	1 = North (N)	6.9	3.1
	2 = Northeast (NE)	21.8	13.5
	3 = Southeast (SE)	46.2	34.6
	4 = South (S)	17.0	11.6
	5 = Central-West (C-W)	8.1	4.3
	Anonymized removed data	---	32.9
Gender / Quasi-identifier	M = Male	44.2	27.9
	F = Female	55.8	39.2
	Anonymized removed data	---	32.9
Race-skin color/Quasi-identifier	A =White	54.5	42.0
	B = Black	7.9	3.0
	C = Yellow	2.5	0.6
	D =Pardo	33.1	21.2
	E =Indigenous	0.3	0.0
	F = Not declared	1.8	0.2
	Anonymized removed data	---	33.0
Father's education/Insensitive	1 = None	4.2	4.2
	2 = 1st – 5th grade	21.8	21.8
	3 = 6th – 9th grade	15.3	15.3
	4 =Secondary school	35.3	35.3
	5 =Graduation	16.9	16.9
	6 = Post-graduation	6.5	6.5
Mother's education/Insensitive	1 = None	2.1	2.1
	2 = 1st – 5th grade	16.2	16.1
	3 = 6th – 9th grade	13.7	13.7
	4 =Secondary school	36.4	36.5
	5 =Graduation	19.6	19.6
	6 = Post-graduation	12.0	12.0
Family's income/Insensitive	1 = Up to 1.5 min. wages	18.0	17.9
	2 = 1.5 to 3 min. wages	27.9	28.0
	3 = 3 to 4.5 min. wages	20.3	20.3
	4 = 4.5 to 6 min. wages	11.5	11.6
	5 = 6 to 10 min. wages	12.3	12.3
	6 = 10 to 30 min. wages	8.3	8.3
	7 = Above 30 min. wages	1.7	1.7
