# Supplementary material for: Subtle biases introduced in equity studies through data anonymization
Source: PLoS One. 2025 Oct 8;20(10):e0332441. doi: 10.1371/journal.pone.0332441 (PMC12507250; doi:10.1371/journal.pone.0332441)
Supplement: S2 Table — (PDF) [file pone.0332441.s002.pdf]

Variable	Modality	Prev_C0	Prev_C1	Prev_C2	Prev_C3	Prev_C4	Prev_C5	Cohort	Lift_C0	Lift_C1	Lift_C2	Lift_C3	Lift_C4	Lift_C5
Gender	F	0,822	0,378	0,137	0,607	0,707	0,720	0,558	1,475	0,678	0,246	1,088	1,268	1,290
	M	0,178	0,622	0,863	0,393	0,293	0,280	0,442	0,401	1,406	1,950	0,889	0,662	0,634
Region	1	0,065	0,066	0,049	0,050	0,059	0,125	0,069	0,941	0,950	0,706	0,723	0,851	1,807
	2	0,120	0,147	0,108	0,161	0,160	0,551	0,218	0,548	0,675	0,496	0,738	0,736	2,531
	3	0,319	0,556	0,641	0,522	0,526	0,196	0,462	0,690	1,204	1,388	1,131	1,140	0,425
	4	0,409	0,168	0,133	0,181	0,175	0,032	0,170	2,404	0,985	0,784	1,066	1,031	0,189
	5	0,088	0,064	0,069	0,086	0,079	0,096	0,081	1,082	0,789	0,851	1,055	0,976	1,173
Income	1	0,185	0,131	0,040	0,000	0,329	0,378	0,180	1,028	0,730	0,224	0,000	1,833	2,104
	2	0,399	0,306	0,191	0,000	0,425	0,408	0,279	1,433	1,099	0,686	0,000	1,525	1,464
	3	0,325	0,259	0,340	0,007	0,174	0,168	0,203	1,598	1,273	1,675	0,034	0,858	0,828
	4	0,091	0,142	0,243	0,122	0,051	0,038	0,115	0,791	1,228	2,108	1,058	0,440	0,333
	5	0,000	0,122	0,160	0,395	0,017	0,007	0,123	0,000	0,986	1,295	3,195	0,139	0,061
	6	0,000	0,038	0,024	0,389	0,003	0,000	0,083	0,000	0,463	0,292	4,703	0,042	0,003
	7	0,000	0,002	0,001	0,087	0,000	0,000	0,017	0,000	0,132	0,045	5,157	0,016	0,000
Father's	1	0,000	0,103	0,006	0,000	0,135	0,028	0,042	0,009	2,470	0,140	0,012	3,234	0,679
education	2	0,018	0,477	0,091	0,010	0,570	0,224	0,218	0,084	2,195	0,418	0,047	2,621	1,030
	3	0,080	0,186	0,187	0,036	0,204	0,217	0,153	0,521	1,211	1,221	0,232	1,331	1,415
	4	0,498	0,197	0,541	0,279	0,086	0,461	0,353	1,411	0,557	1,532	0,791	0,245	1,305
	5	0,311	0,035	0,157	0,419	0,004	0,066	0,169	1,835	0,204	0,928	2,476	0,027	0,390
	6	0,092	0,003	0,018	0,255	0,000	0,004	0,065	1,419	0,042	0,272	3,927	0,000	0,057
Mother's	1	0,000	0,073	0,001	0,000	0,074	0,001	0,021	0,000	3,495	0,025	0,004	3,550	0,034
education	2	0,005	0,458	0,033	0,004	0,550	0,044	0,162	0,030	2,823	0,201	0,024	3,387	0,271
	3	0,048	0,217	0,134	0,021	0,259	0,155	0,137	0,353	1,585	0,981	0,157	1,893	1,131
	4	0,439	0,220	0,537	0,228	0,112	0,577	0,364	1,207	0,604	1,476	0,627	0,306	1,586
	5	0,308	0,028	0,219	0,405	0,006	0,155	0,196	1,572	0,144	1,119	2,068	0,030	0,788
	6	0,199	0,004	0,076	0,341	0,000	0,069	0,120	1,659	0,036	0,635	2,839	0,000	0,575
Race	A	0,812	0,487	0,665	0,732	0,539	0,077	0,545	1,491	0,894	1,221	1,344	0,990	0,142
	B	0,050	0,104	0,077	0,033	0,107	0,104	0,079	0,641	1,326	0,983	0,424	1,365	1,317
	C	0,023	0,023	0,023	0,027	0,025	0,026	0,025	0,915	0,914	0,950	1,105	0,998	1,050
	D	0,095	0,364	0,210	0,184	0,310	0,775	0,331	0,287	1,099	0,634	0,557	0,937	2,343
	E	0,002	0,004	0,003	0,001	0,004	0,004	0,003	0,719	1,292	0,963	0,491	1,315	1,285
	F	0,018	0,018	0,021	0,022	0,014	0,014	0,018	1,000	1,016	1,184	1,204	0,806	0,785
