# Supplementary material for: Subtle biases introduced in equity studies through data anonymization
Source: PLoS One. 2025 Oct 8;20(10):e0332441. doi: 10.1371/journal.pone.0332441 (PMC12507250; doi:10.1371/journal.pone.0332441)
Supplement: S3 Table — (PDF) [file pone.0332441.s003.pdf]

Variable	Modality	Prev_C0	Prev_C1	Prev_C2	Prev_C3	Prev_C4	Prev_C5	Cohort	Lift_C0	Lift_C1	Lift_C2	Lift_C3	Lift_C4	Lift_C5
Gender	F	0,859	0,551	0,066	0,653	0,668	0,714	0,585	1,469	0,943	0,113	1,117	1,142	1,221
	M	0,141	0,449	0,934	0,347	0,332	0,286	0,415	0,340	1,080	2,247	0,835	0,800	0,689
Region	1	0,032	0,005	0,033	0,034	0,031	0,116	0,046	0,701	0,109	0,714	0,743	0,689	2,546
	2	0,084	0,072	0,111	0,150	0,139	0,605	0,202	0,417	0,355	0,550	0,741	0,691	2,997
	3	0,634	0,812	0,586	0,536	0,561	0,144	0,516	1,228	1,574	1,135	1,039	1,087	0,279
	4	0,192	0,111	0,214	0,210	0,208	0,040	0,172	1,115	0,643	1,241	1,216	1,209	0,232
	5	0,058	0,000	0,057	0,071	0,060	0,095	0,064	0,901	0,004	0,889	1,104	0,933	1,477
Income	1	0,196	0,126	0,005	0,000	0,299	0,376	0,163	1,200	0,770	0,032	0,000	1,832	2,309
	2	0,397	0,321	0,151	0,000	0,423	0,405	0,269	1,476	1,193	0,561	0,000	1,575	1,505
	3	0,319	0,268	0,314	0,007	0,189	0,164	0,203	1,572	1,323	1,545	0,034	0,933	0,808
	4	0,088	0,142	0,255	0,123	0,063	0,046	0,120	0,736	1,187	2,126	1,027	0,529	0,379
	5	0,000	0,111	0,223	0,369	0,022	0,009	0,131	0,001	0,847	1,710	2,824	0,166	0,067
	6	0,000	0,029	0,050	0,404	0,003	0,000	0,094	0,000	0,311	0,533	4,286	0,034	0,004
	7	0,000	0,003	0,002	0,097	0,000	0,000	0,020	0,000	0,143	0,084	4,790	0,016	0,000
Father's	1	0,000	0,038	0,005	0,000	0,103	0,031	0,026	0,007	1,467	0,205	0,008	3,972	1,175
education	2	0,018	0,338	0,081	0,006	0,597	0,245	0,183	0,098	1,851	0,443	0,035	3,268	1,343
	3	0,115	0,210	0,171	0,029	0,222	0,241	0,154	0,749	1,368	1,114	0,191	1,440	1,569
	4	0,575	0,330	0,520	0,249	0,078	0,417	0,373	1,541	0,884	1,395	0,667	0,209	1,117
	5	0,238	0,072	0,195	0,433	0,001	0,061	0,190	1,255	0,381	1,030	2,285	0,003	0,323
	6	0,054	0,011	0,027	0,282	0,000	0,005	0,075	0,716	0,152	0,359	3,763	0,000	0,064
Mother's	1	0,000	0,020	0,000	0,000	0,045	0,000	0,009	0,004	2,262	0,047	0,004	5,077	0,045
education	2	0,010	0,296	0,026	0,002	0,545	0,039	0,122	0,078	2,415	0,211	0,020	4,455	0,318
	3	0,093	0,219	0,111	0,016	0,276	0,150	0,129	0,723	1,703	0,864	0,126	2,148	1,169
	4	0,532	0,373	0,478	0,196	0,131	0,553	0,380	1,399	0,982	1,259	0,515	0,344	1,455
	5	0,250	0,071	0,269	0,413	0,003	0,168	0,222	1,130	0,320	1,212	1,864	0,013	0,760
	6	0,115	0,022	0,116	0,373	0,000	0,089	0,139	0,832	0,156	0,834	2,685	0,000	0,643
Race	A	0,786	0,694	0,740	0,795	0,630	0,085	0,626	1,255	1,109	1,182	1,271	1,006	0,136
	B	0,048	0,012	0,039	0,020	0,064	0,069	0,045	1,070	0,259	0,870	0,435	1,419	1,534
	C	0,012	0,000	0,006	0,012	0,009	0,009	0,009	1,294	0,000	0,649	1,325	0,974	1,000
	D	0,149	0,294	0,211	0,168	0,294	0,835	0,316	0,472	0,929	0,666	0,531	0,930	2,639
	E	0,000	0,000	0,000	0,000	0,000	0,000	0,000	0,000	0,000	0,000	0,000	0,000	0,000
	F	0,005	0,000	0,004	0,005	0,003	0,002	0,004	1,347	0,000	1,148	1,336	0,812	0,507
